# Testicular mitochondrial redox imbalance and impaired oxidative phosphorylation underlie microplastic-induced testicular dysfunction in Wistar rats

**DOI:** 10.3389/ftox.2026.1699288

**Published:** 2026-01-15

**Authors:** Samuel Abiodun Kehinde, Tolulope Peter Fatokun, Sarva Mangala Praveena, Abosede Temitope Olajide, Chau Ling Tham, Mariana Teles Pereira, Sasitorn Chusri

**Affiliations:** 1 Biomedical Technology Research Group for Vulnerable Populations and School of Health Science, Mae Fah Luang University, Chiang Rai, Thailand; 2 Biochemical Toxicology Laboratory, Department of Environmental Health Sciences, Faculty of Basic Medical Sciences, Ajayi Crowther University, Oyo, Nigeria; 3 Department of Drug Toxicology and Safety Pharmacology, Faculty of Life Sciences, University of Bradford, Bradford, United Kingdom; 4 Department of Environmental and Occupational Health, Faculty of Medicine and Health Sciences, Universiti Putra Malaysia, Serdang, Malaysia; 5 Cell and Signaling Laboratory, Department of Biomedical Science, Faculty of Medicine and Health Sciences, Universiti Putra Malaysia (UPM), Serdang, Malaysia; 6 Department of Cell Biology, Physiology and Immunology, Universitat Autònoma de Barcelona, Barcelona, Spain

**Keywords:** electron transport chain, microplastics, mitochondrial dysfunction, oxidative phosphorylation, polyethylene microplastics, redox imbalance, reproductive toxicity

## Abstract

Polyethylene microplastics (PE-MPs), now pervasive environmental contaminants, have been implicated in reproductive toxicity, but their mechanistic effects on testicular function remain poorly defined. This study investigates the mechanistic basis of PE-MPs–induced male reproductive toxicity in a rodent model (Wistar rats), with a specific focus on testicular mitochondrial redox homeostasis and oxidative phosphorylation. By integrating mitochondrial bioenergetics, redox signaling, histopathology, and reproductive endpoints, the work advances mechanistic toxicology insights relevant to environmental reproductive health. Fifteen male rats were randomly divided into three groups: control, and PE-MPs treated groups receiving 15 or 60 mg/kg body weight orally for 28 days. Testicular mitochondria were isolated to evaluate activities of tricarboxylic acid (TCA) cycle enzymes, citrate synthase (CS), isocitrate dehydrogenase (IDH), succinate dehydrogenase (SDH), and malate dehydrogenase (MDH), as well as respiratory chain complexes I–IV. Mitochondrial redox balance indices, including malondialdehyde (MDA), myeloperoxidase (MPO), reduced glutathione (GSH), catalase (CAT), and superoxide dismutase (SOD), were also assessed. PE-MP exposure induced a dose-dependent suppression of TCA cycle and electron transport activities, with CS and SDH inhibited by up to 50% at the highest dose suggesting a broad inhibition of electron transport and ATP synthesis. These mitochondrial impairments coincided with elevated MDA and MPO levels, and significant depletion of GSH, CAT, and SOD, indicating profound mitochondrial oxidative distress. These mitochondrial disturbances correlated with histological evidence of testicular degeneration and decreased testosterone levels. Collectively, the findings of this study highlight that PE-MPs compromise testicular bioenergetics and function by disrupting testicular oxidative phosphorylation and redox homeostasis, leading to mitochondrial dysfunction, structural degeneration, and impaired steroidogenesis, providing mechanistic insight into microplastic-induced male infertility. Understanding this bioenergetic collapse provides a biochemical framework for assessing the reproductive toxicity of microplastics and underscores the urgency of mitigating their exposure in vulnerable populations.

## 1 Introduction

Microplastics (MPs), including polyethylene microplastics (PE-MPs), are now pervasive environmental contaminants arising from the degradation of plastic materials used globally in packaging and consumer products (De Souza Machado et al., 2018; Dubey et al., 2022; Yong et al., 2020; Campanale et al., 2020). PE-MPs, widely derived from packaging and consumer products, are among the most frequently detected polymer types in food, water, air, and even human blood and stool (Yong et al., 2020; Campanale et al., 2020). Owing to their small size, chemical persistence, and hydrophobic surfaces, MPs can traverse biological barriers, enter systemic circulation, and accumulate in metabolically active organs, including the liver, kidney, brain, and reproductive tissues (Garcia et al., 2023; Lu et al., 2023; Urli et al., 2023; Enyoh et al., 2023; Kehinde et al., 2024). Among polymer types, polyethylene is one of the most abundant and environmentally persistent, yet its tissue-specific toxicodynamics remain less well defined than those of polystyrene or nanoplastics and chronic accumulation of PE-MPs in living organisms has shifted scientific attention from ecological toxicity to human and animal health, particularly in relation to endocrine and reproductive functions (Urli et al., 2023).

The testes are uniquely vulnerable to mitochondrial toxicants due to their exceptionally high bioenergetic demands. Spermatogenesis requires continuous ATP generation to support germ cell proliferation, meiosis, flagellar assembly, and chromatin remodeling, processes that are heavily dependent on mitochondrial oxidative phosphorylation (Aitken et al., 2012; Aitken, 2017). In Leydig cells, mitochondrial integrity is essential for cholesterol transport and steroidogenesis via the steroidogenic acute regulatory (StAR) protein, making mitochondrial dysfunction a direct threat to testosterone biosynthesis (Zhou et al., 2023; Ojo et al., 2025). Furthermore, spermatozoa possess membranes enriched in polyunsaturated fatty acids and limited antioxidant capacity, rendering them particularly susceptible to mitochondrial ROS-mediated lipid peroxidation (Aitken et al., 2012; Aitken and Baker, 2020).

Despite increasing recognition of microplastic-induced reproductive toxicity, critical mechanistic gaps remain regarding polyethylene microplastics specifically. Most *in vivo* studies have focused on polystyrene particles or nanoplastics, while PE-MPs differ substantially in surface chemistry, hydrophobicity, additive profiles, and environmental prevalence (Dubey et al., 2022; Campanale et al., 2020). Moreover, whether PE-MPs directly impair testicular mitochondrial bioenergetics, particularly oxidative phosphorylation and redox balance has not been systematically examined *in vivo*. The present study addresses this gap by providing a focused and comprehensive analysis of how PE-MP exposure disrupts testicular oxidative phosphorylation, mitochondrial redox homeostasis, and biochemical markers of testicular function in Wistar rats by assessing dose-dependent alterations in key enzymes of the TCA cycle and electron transport chain, alongside oxidative stress markers and histological evaluations.

## 2 Materials and methods

### 2.1 Chemicals, kits and reagents

PE-MPs were sourced from Sigma-Aldrich (34–50 μm; Product no: 434272; Batch No: MKCV4850; CAS no. 9002-88-4) and used without further chemical modification. According to the supplier CoA, the particles are polyethylene (≥98% purity) with a nominal particle size distribution that conforms to 42 μm, consist of irregularly shaped fragments rather than perfectly spherical particles, a morphology commonly reported for environmentally relevant polyethylene microplastics (Certificate of Analysis (CoA) on file). All other reagents used were of analytical grade, obtained from Sigma (St. Louis, MO, United States) and Carl Roth GmbH, Karlsruhe, Germany.

### 2.2 Animals and experimental design

Fifteen male Wistar rats (180–200 g) of 8 weeks old were kept in the animal facility at Ajayi Crowther University for a 7-day acclimatization period before exposure to PE-MPs. Housed in standard cages, the animals were given clean drinking water and a consistent crumble diet manufactured by Vita Feeds Nigeria Limited. Throughout the course of the study, environmental conditions, temperature of 22 °C ± 2 °C, 12-h light and dark cycle of 06:00–18:00 h were kept. The rats were assigned into three groups of five rats each and received treatments as follows: group 1 (control) rats received normal saline orally for 28 days; group 2 and 3 received 15 and 60 mg/kg PE-MPs orally (gavage), respectively. Animals were randomly allocated to experimental groups using a simple randomization procedure. Investigators performing sperm analysis, histological evaluation, and biochemical assays were blinded to treatment groups to minimize observer bias.

Prior to dosing, the required amount of PE-MPs was weighed gravimetrically on an analytical balance (±0.1 mg) to prepare each dose (15 or 60 mg/kg body weight). For administration, PE-MPs were suspended in a standardized vehicle to ensure a stable and homogeneous dispersion suitable for oral gavage. The vehicle consisted of 0.5% (w/v) CMC in sterile normal saline, supplemented with 0.02% (v/v) Tween-80 to improve wetting and minimize particle aggregation. The vehicle was prepared fresh and autoclaved before use. For each dosing session, PE-MPs were added to the vehicle and pre-wetted, followed by bath sonication for 10 min to disrupt aggregates. The suspension was kept on ice and gently vortexed immediately prior to each gavage. Final gavage volume was 1 mL/100 g body weight (i.e., approximately 1.8–2.0 mL per rat; exact volume adjusted to individual body weight on dosing day). Suspensions were visually inspected for large aggregates before dosing and gently re-suspended between animals to minimise settling. Gravimetric checks (weigh back of aliquots prepared for dosing) were performed to confirm the delivered mass per gavage (mean recoveries within ±5% of target mass). Animals were observed twice daily for clinical signs (general appearance, behaviour, respiratory rate, signs of regurgitation or vomiting, and mortality) and body weights were recorded weekly. No mortality, evidence of regurgitation/vomiting, or other overt adverse clinical signs were observed during the 28-day exposure period. Doses (15 and 60 mg/kg) were selected to match previously published sub-chronic PE-MP exposure regimens (Enyoh et al., 2023; Farag et al., 2023; Fernández-Vizarra et al., 2010) and to provide a low and a high exposure level for mechanistic evaluation. As requested by the National Research Council (Guide for the Care and Use of Laboratory Animals, 2010), all animal-related activities followed internationally recognized standards for the care and use of laboratory animals and the experimental protocol was reviewed and approved by the Ethical Review Committee of Ajayi Crowther University, under reference number FNS/ERC/2024/019TD to ensure compliance with national legislation and the ARRIVE guidelines.

### 2.3 Organs sampling and biochemical assays

Following an overnight fast after 28 days of exposure to PE-MPs, the animals were sacrificed under mild xylazine/ketamine anaesthesia (100 mg ketamine/kg, 10 mg xylazine/kg) cocktail intraperitoneally before blood sampling via cardiac puncture, and this was followed by organ excision (testes and epididymis). After being extracted, the testis, epididymis and seminal vesicles were removed and their weights measured, and the testis weight index was calculated. The testes were rinsed in 1.15% ice-cold KCl and then homogenized in 5 volume/weight of ice-cold 0.1M phosphate buffer (pH 7.4). For mitochondrial enzyme assays, testicular mitochondria were isolated by differential centrifugation as described by Fernández-Vizarra et al. (Fernández-Vizarra et al., 2010). The post-mitochondrial supernatant was reserved for non-mitochondrial biochemical assays. Total protein concentration in the testes was measured at 540 nm using the Biuret method, following the protocol described by Gornall et al. (1949).

### 2.4 Semen collection and microscopy analysis

Sperm cells were isolated surgically from the epididymis of the left testis, and the semen was obtained through the cauda epididymis. The cauda epididymis was pierced with a small incision, and sperm cells were aspirated into a Pasteur pipette. The epididymal tract was then washed with 2–3 drops of 2.9% buffered sodium citrate at physiological temperature (37 ^O^C). The specimen collected was divided into two-halves; one-half was stained with Wells and Awa stains to analyze morphologically, whereas the other half was combined with 0.5 mL of 2.9% sodium citrate to determine progressive motility. Mass activity was determined by studying the undiluted specimen. Progressive motility and live-dead ratio were determined under high-power magnification, and mass activity was determined using an eyepiece graticule (Ow et al., 2020).

### 2.5 Testicular oxidative phosphorylation enzymes activities

Testicular mitochondrial functioning and metabolic activity were assessed by conventional spectrophotometric enzyme assay methods. All enzyme activities were normalized to mitochondrial protein content and expressed as specific activity per milligram of protein. The activity of citrate synthase (CS) was measured by following the method of Nulton-Persson and Szweda (Nulton-Persson and Szweda, 2001) which spectrophometrically at 412 nm measured CoA-SH released using DTNB to form TNB. Isocitrate dehydrogenase (IDH) was assayed spectrophotometrically at 340 nm according to the protocol described by Romkina and Kiriukhin (Romkina and Kiriukhin, 2017) following the principle of NADP + reduction to NADPH. Malate dehydrogenase (MDH) activity was evaluated reduction of oxaloacetate coupled to oxidation of NADH according to a method utilized by López-Calcagno et al. (2009) and succinate dehydrogenase (SDH) activity was measured via DCIP reduction at 600 nm in the presence of decy-ubiquinone, a method described by Jones and Hirst (2013). In addition, we investigated the functional capacity of the mitochondrial through the assessment of electron transport chain (ETC) by quantifying the activities of the key respiratory chain complexes; NADH ubiquinone oxidoreductase (NADH oxidation at 340 nm, reported as rotenone-sensitive activity), succinate ubiquinone oxidoreductase (Succinate-driven reduction of DB (dichloroindophenol) via decyl-ubiquinone monitored at 600 nm), cytochrome c oxidoreductase (Reduction of cytochrome c measured at 550 nm) and cytochrome c oxidase (Oxidation of reduced cytochrome c monitored at 550 nm) using the protocol described by Medja et al. (2009) These assays were carried out using the isolated mitochondria isolate from the testicular tissue based on the detailed protocol provided by Fernández-Vizarra et al. (2010) using differential centrifugation, ice-cold isolation buffer (225 mM mannitol, 75 mM sucrose, 5 mM HEPES, 1 mM EGTA, pH 7.4), and all steps performed at 4 °C.

### 2.6 Testicular mitochondrial redox and inflammatory status

Mitochondrial oxidative stress and antioxidant status were evaluated in testicular tissue. Oxidative stress and inflammatory markers were quantified in isolated mitochondrial fractions unless otherwise stated. The level of malondialdehyde (MDA), a well-known indicator of lipid peroxidation was quantified using the procedure of Devasagay et al. (2003) which has its principle based on MDA reaction with Thiobarbituric acid (TBA) to form a pink MDA-TBA adduct measurable at 532 nm. Nitric oxide (NO) concentration was measured using the Griess reagent, as described by Guevara et al. (1998) in which nitrite, the stable metabolite of NO, reacts with Griess reagent to form a purple azo dye measured at 540 nm. Although myeloperoxidase is classically associated with neutrophils, MPO activity in testicular tissue has been used as an indicator of local inflammatory oxidative stress and has been linked to impaired spermatogenesis and reduced sperm quality (Hasan et al., 2022), the myeloperoxidase (MPO) activity was evaluated spectrophotometrically using the protocol outlined by Hanning et al. (2023) in which MPO catalyses oxidation of 0-dianisidine by hydrogen peroxide and product absorbance measured at 460 nm. The levels of reduced glutathione (GSH) were estimated according to Moron et al. (1979) methodology in which GSH reacts with DTNB to form yellow TNB measured at 4112 nm and glutathione S-transferase (GST) activity was determined based on the ability of GST to catalyse conjugation of GSH with CDNB and product monitored at 340 nm as described by a protocol provided of Skopelitou and Labrou (Skopelitou and Labrou, 2010). The activities of the antioxidant enzyme superoxide dismutase (SOD) was based on the potential inhibition of autoxidation of epinephrine to adrenochrome by SOD and monitored at 480 nm under alkaline conditions as described by Misra and Fridovich (1972) and catalase (CAT) were quantified according to the procedure described in and in Hadwan (2018) in which CAT decomposes hydrogen peroxide while the remaining hydrogen peroxide reacts with ammonium molybdate to give a yellow complex measured at 374 nm.

### 2.7 Sperm parameters and testosterone level assessment

Sperm viability was determined using the eosin-nigrosin staining method reported by Björndahl et al. (2003), with slight modifications. Viability was determined using eosin Y staining (5% in saline). A glass slide containing 40 uL of freshly prepared sperm suspension was combined with 10 μL eosin and examined under a light microscope (×400 magnification). After staining, live sperms remained unstained, while those with any pink or red coloration were identified as dead. At least 200 sperm were counted from each sample in 10 randomly selected fields of vision, and the proportion of viable sperms was recorded.

To determine sperm motility, semen was re-suspended and a 40 μL aliquot of freshly liquefied semen was placed on a glass slide maintained at 37 °C and recorded using a video microscope (Olympus BX51, Germany). Ten random fields per slide were captured and motility was scored visually according to the progressive, non-progressive and immotile classification following (Björndahl et al., 2003; Cheng et al., 2006) (progressive/non-progressive/immotile on the 1–2 scale used in those references). The percentage motile reported in Table 2 therefore derives from scoring ≥10 fields per sample.

Diff-Quick staining was used to determine the morphology of spermatozoa (World Health Organization, 1999). Cauda epididymis was minced in 1 mL of normal saline solution at 37 °C. One drop of this preparation was put on a microscope slide, fixed in fixative solution (1.8 mg/L triarylmethane in methyl alcohol) for 15 s, stained in solution No. 1 (1 g/L xanthene in sodium azide-preserved buffer) for 10 s and in solution No. 2 (1.25 g/L thiazine dye mixture, 0.625 g/L Azure A and 0.625 g/L methylene blue in buffer) for 5 s and dried at room temperature. Slides were then mounted and observed under light microscope at 1000x magnification. Any deformities of the tail, neck or head were noted. The levels of testosterone in the serum were determined in all experimental groups by following the manufacturer’s procedure as described by the testosterone assay kit (CAT 70619) from BioCheck Inc., United States.

### 2.8 Histopathological analysis of the testicular tissue

Histological evaluation was performed in a blinded manner. Seminiferous tubule integrity was additionally assessed using the Johnsen scoring system (Johnsen, 1970). The testes removed was fixed by immersing it in 10% formalin for 48 h. Following tissue processing, a rotary microtome (Leica Model RM 2145, Germany) was used to create sections that were 5 µm thick. A camera-equipped Leica DM750 microscope was then used by the histopathologist to view the slides for histology after they had been stained with hematoxylin and eosin (H&E). The histological investigation looked at the quantity of spermatogonia, primary spermatocytes, spermatids, and Leydig cells.

### 2.9 Statistical analysis

Data were screened for outliers using boxplots and z-scores (>3 SD). Normality was assessed with the Shapiro–Wilk test and homogeneity of variances with Levene’s test. The Statistical Package for the Social Sciences software, version 16.0 for Windows (SPSS Inc., Chicago, II, United States), was used to perform the statistical analysis employing one-way analysis of variance (ANOVA), followed by Tukey’s *post hoc* multiple comparison test to assess intergroup differences. Means ± standard deviation (SD) was used to present all data. The threshold for statistical significance was set at P < 0.05.

## 3 Results

### 3.1 Testicular mitochondrial oxidative phosphorylation disruption

Exposure to PE-MPs significantly altered the activities of key enzymes involved in testicular mitochondrial oxidative phosphorylation, indicating dose-dependent mitochondrial dysfunction.

- TCA Cycle Enzyme Activities

Mitochondrial citrate synthase (CS) activity was significantly reduced in rats exposed to 15 mg/kg and 60 mg/kg PE-MPs. Compared to controls, CS activity dropped by 26.3% at 15 mg/kg (P < 0.05) and by 41.5% at 60 mg/kg (P < 0.05). Similarly, IDH showed a progressive decline: rats exposed to 15 mg/kg exhibited a 22.6% reduction, while 60 mg/kg exposure led to a 37.9% decrease relative to control (P < 0.05 for both). SDH activity declined by 29.4% at 15 mg/kg and by a more pronounced 47.1% at 60 mg/kg (P < 0.05), pointing to impaired electron entry into the mitochondrial respiratory chain via Complex II. In contrast, MDH activity was not perturbed significantly at both 15 and 60 mg/kg BW.

- Electron Transport Chain (ETC) Complex Activities

**FIGURE 1:**
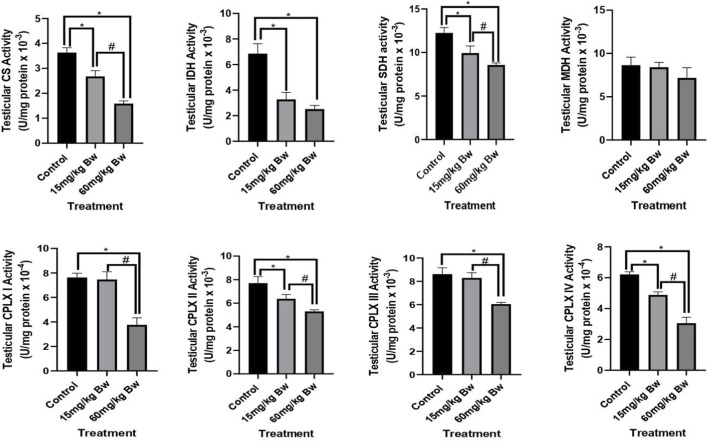
Testicular oxidative phosphorylation enzyme activities in rats exposed to PE-MPs. The values are presented as mean ± SD (n = 5) per group. *Significantly (*P* < 0.05) different when compared with control; # significantly (*P* < 0.05) different relative to 15 mg/kg PE-MPs. CS- Citrate Synthase, IDH-Isocitrate Dehydrogenase, SDH- Succinate Dehydrogenase, MDH- Malate Dehydrogenase, Complex I-CPLX I, Complex II- CPLXII, Complex III-CPLX III, Complex IV-CPLX IV.

Polyethylene microplastics exposure also significantly compromised the activity of key, ETC complexes (Figure 1). Complex I (CPLX I) activity was diminished by 25.8% and 44.2% in the 15 mg/kg and 60 mg/kg groups, respectively (P < 0.05), complex II (CPLX II) followed a similar trend, decreasing by 30.6% and 48.4% (P < 0.05), further supporting the inhibition of succinate-dependent electron flow. At 60 mg/kg PE-MPs, the rats showed the most severe reductions across the, ETC, with Complex III (CPLX III) activity decreasing by 42.7% and complex IV (CPLX IV) dropping by 46.8% compared to control (P < 0.05). Notably, significant differences were also observed between the 15 mg/kg and 60 mg/kg groups for these complexes (P < 0.05), emphasizing a clear dose–response effect. Taken together, these data suggest subacute exposure to PE-MPs leads to a marked, dose-dependent impairment of both TCA cycle enzymes and mitochondrial electron transport chain complexes in testicular tissue.

### 3.2 Testicular mitochondrial redox status in rats exposed to PE-MPs

Exposure to PE-MPs significantly disrupted mitochondrial redox balance in a dose-dependent manner (Table 1). This was characterized by reduced antioxidant enzyme activities and increased oxidative and nitrosative stress markers.

- Enzymatic and non-enzymatic antioxidant reductions

**TABLE 1:** Testicular Mitochondrial redox status in rats exposed to PE-MPs (n = 5 rats per group; values expressed as mean ± SD).

Biomarker	Control	15 mg/kg PE-MPs	60 mg/kg PE-MPs
Catalase (CAT) U/mg protein	53.70 ± 3.20	43.30 ± 2.70^*^	29.70 ± 2.20^*#^
Glutathione (GSH) µmol/g tissue	6.20 ± 0.20	5.70 ± 0.30^*^	3.80 ± 0.60^*#^
Glutathione S-transferase (GST) U/mg protein	24.30 ± 1.70	17.40 ± 1.50^*^	14.10 ± 1.20^*#^
Superoxide dismutase (SOD) U/mg protein	26.70 ± 2.20	21.10 ± 1.70^*^	15.30 ± 1.70^*#^
Malondialdehyde (MDA) nmol/mg protein	1.26 ± 0.06	1.78 ± 0.12^*^	3.15 ± 0.24^*#^
Myeloperoxidase (MPO) U/mg protein	1.21 ± 0.12	1.70 ± 0.20^*^	2.84 ± 0.22^*#^
Nitric oxide (NO) µmol/g tissue	5.90 ± 0.20	7.42 ± 0.40^*^	9.20 ± 0.60^*#^

^*^Significantly (P < 0.05) different when compared with control; # significantly (P < 0.05) different when compared with 15 mg/kg PE-MPs.

The activities of key antioxidant enzymes, including CAT, GST, and SOD, were significantly suppressed following exposure to PE-MPs. At 15 mg/kg, CAT activity declined by approximately 19%, and this suppression deepened to about 45% at 60 mg/kg. GST showed a comparable pattern, decreasing by 28% at the lower dose and by over 40% at the higher dose. SOD activity followed a similar trend, with reductions of 21% and 43%, respectively. These findings suggest a compromised ability of testicular tissue to neutralize ROS, particularly at higher exposure levels. Reduced glutathione was also significantly depleted in a dose-dependent fashion. While the 15 mg/kg group showed a mild (8%) reduction, the 60 mg/kg group experienced a sharp 39% decline compared to controls. This depletion suggests a diminished cellular defense capacity against oxidative insults induced by PE-MPs.

- Elevated Oxidative and Nitrosative Stress Markers

Corroborating the antioxidant decline, levels of MDA, a by-product of lipid peroxidation, were significantly elevated. MDA increased by approximately 41% at 15 mg/kg and surged by over 250% at 60 mg/kg, highlighting extensive lipid membrane damage due to oxidative stress. Similarly, MPO, a pro-oxidant enzyme linked to inflammation and ROS generation, rose by 40% and 135% in the 15 mg/kg and 60 mg/kg groups, respectively. The increase in MPO activity supports the involvement of inflammatory oxidative cascades in PE-MP toxicity.

Nitric oxide levels, another oxidative stress marker with known cytotoxic potential at high concentrations, also rose significantly in a dose-dependent manner. The 15 mg/kg dose resulted in a 26% increase, whereas the 60 mg/kg group showed an over 55% elevation compared to controls. These changes suggest an imbalance between NO production and detoxification, contributing further to testicular redox disruption.

### 3.3 Comparative analysis of sperm parameters in rats exposed to PE-MPs

Exposure to PE-MPs induced marked and dose-dependent reproductive toxicity in male Wistar rats, as evidenced by alterations in sperm quality, morphology, reproductive organ weights, and serum testosterone levels. Table 2 shows the effects of PE-MPs at two doses (15 mg/kg and 60 mg/kg body weight) on various reproductive parameters in male Wistar rats.

- Sperm Viability and Motility Decline with PE-MP Exposure

**TABLE 2:** Sperm function and morphological changes following pe-mps exposure in rats. (*n* = 5 rats per group; values expressed as mean ± SD).

Parameter	Control	15 mg/kg PE-MPs	60 mg/kg PE-MPs
Sperm livability (%)	91.4 ± 2.3	72.2 ± 4.6^*^	52.6 ± 6.4^*#^
Sperm motility (%)	88.1 ± 3.5	64.5 ± 5.7^*^	38.4 ± 6.8^*#^
Sperm concentration (×10^6^/mL)	86.3 ± 5.8	43.7 ± 7.5^*^	23.8 ± 4.6^*#^
Normal morphology (%)	90.2 ± 2.4	61.3 ± 4.1^*^	39.5 ± 6.7^*#^
Free head (%)	2.3 ± 0.6	8.6 ± 1.7^*^	8.3 ± 1.9^*^
Free tail (%)	2.6 ± 0.5	8.7 ± 1.3^*^	14.9 ± 1.9^*#^
Coiled tail (%)	1.6 ± 0.4	7.5 ± 1.7^*^	7.2 ± 1.9^*^
Bent tail (%)	1.6 ± 0.4	7.1 ± 1.0^*^	10.3 ± 1.7^*#^
Dwarf tail (%)	0.9 ± 0.3	3.9 ± 0.6^*^	3.7 ± 0.7^*^
Mid piece bent (%)	0.7 ± 0.2	0.7 ± 0.6	0.8 ± 0.8
Acrosomal defect (%)	0.8 ± 0.4	3.5 ± 0.5^*^	4.9 ± 0.4^*#^
Total abnormal (%)	10.8 ± 2.8	35.7 ± 5.1	63.5 ± 6.8^*#^
Total normal (%)	95.7 ± 3.4	66.3 ± 7.1^*^	38.5 ± 6.3^*#^
Testosterone (ng/mL)	5.9 ± 0.5	2.4 ± 0.4^*^	1.3 ± 0.2^*#^
Testis weight (g)	1.64 ± 0.05	1.21 ± 0.06	0.98 ± 0.20^*#^
Epididymis weight (g)	0.60 ± 0.02	0.37 ± 0.07^*^	0.32 ± 0.02^*^
Seminal vesicle weight (g)	0.72 ± 0.02	0.55 ± 0.04^*^	0.31 ± 0.02^*#^

*Significantly (P < 0.05) different when compared with control; # significantly (P < 0.05) different when compared with 15 mg/kg PE-MPs.

Exposure to PE-MPs significantly compromised sperm functional integrity in a dose-dependent fashion (P < 0.05). Sperm viability in the highest dose group (60 mg/kg) was reduced by approximately 42% compared to the control, while motility declined by over 60%. Even at the lower dose (15 mg/kg), both parameters showed significant reductions, reflecting early signs of functional sperm impairment.

- Reduced Sperm Concentration and Morphological Integrity

A pronounced decline in sperm concentration was observed, with a nearly 73% reduction at the highest PE-MP dose. Similarly, morphologically normal spermatozoa were substantially diminished. Rats exposed to 60 mg/kg of PE-MPs exhibited a 56% reduction in normal morphology compared to controls, while the 15 mg/kg group showed a moderate 32% decline. These structural impairments suggest disrupted spermatogenesis and maturation processes.

- Marked Increase in Sperm Abnormalities

Tail-related defects, including free, coiled, bent, and dwarf tails, were notably elevated. Free tail frequency increased by more than fivefold at the highest dose. Coiled and bent tails surged more than four-fold and six-fold, respectively, relative to controls. Likewise, acrosomal defects, crucial for oocyte penetration, increased six-fold in the 60 mg/kg group. Mid-piece abnormalities remained relatively unchanged, indicating the specificity of PE-MP toxicity to certain tail and head regions. Overall, total abnormal sperm increased nearly six-fold in the high-dose group, while the percentage of total normal sperm plummeted by about 60%, signifying compromised reproductive potential.

- Testosterone Suppression and Organ Atrophy

Testosterone levels were profoundly suppressed by PE-MPs in a dose-responsive manner (P < 0.05). Serum testosterone dropped by approximately 59% in the 15 mg/kg group and nearly 78% in the 60 mg/kg group, indicating endocrine disruption likely contributing to impaired spermatogenesis. Corroborating this hormonal suppression, the weights of reproductive organs testes, epididymis, and seminal vesicles were all significantly reduced. Testicular weight dropped by around 40% at the highest dose, while epididymal and seminal vesicle weights were diminished by approximately 47% and 57%, respectively. These organ-specific reductions reflect potential atrophy and impaired reproductive physiology associated with chronic PE-MP ingestion. Collectively, these findings demonstrate that sub-chronic oral exposure to PE-MPs results in significant and dose-dependent reproductive toxicity in male rats, characterized by reduced sperm quality and quantity, increased morphological abnormalities, decreased reproductive organ weights, and suppressed testosterone production.

### 3.4 PE-MPs distorts testicular histoarchitecture

**FIGURE 2:**
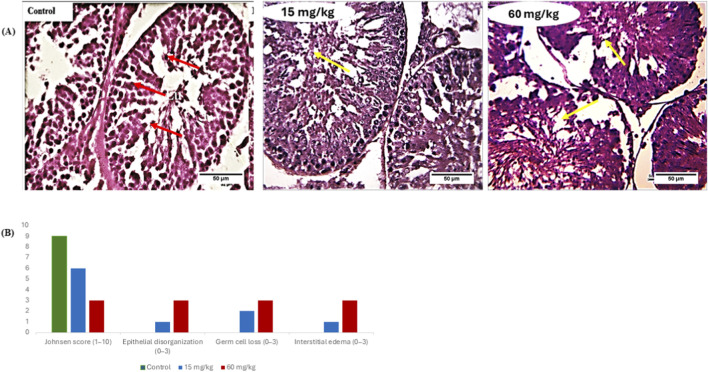
**(A)** Representative photomicrographs of H&E-stained testicular sections from control and PE MPS-treated rats (X400). Control group showing well-organized seminiferous tubules with intact germinal epithelium and active spermatogenesis (red arrows); 15 mg/kg PE-MPs group showing mild-moderate epithelial disorganization, focal germ cell loss, and partial disruption of spermatogenic layers (yellow arrows); 60 mg/kg PE-MPs group showing severe degeneration of seminiferous tubules, marked germ cell depletion, tubular disorganization, and interstitial widening/edema. **(B)** Semi-quantitative histopathology scores derived from blinded evaluation depicting Johnsen score (scale 1–10), epithelial disorganization (scale 0–3), germ cell loss (scale 0–3) and interstitial edema (scale 0–3). The highest damage scores were observed in the 60 mg/kg group, consistent with the photomicrograph findings.

The testicular histoarchitecture is depicted in Figure 2. The control testis displays (red arrow) well-organized seminiferous tubules with active spermatogenesis and intact interstitial tissue. The 15 mg/kg group shows (yellow arrow) mild–moderate epithelial disruption and germ cell loss; some spermatids remain, but layering is disturbed. The 60 mg/kg group reveals a significant reduction in Johnsen score (p < 0.05) of seminiferous tubule architecture, marked loss of germ cells, almost no mature spermatids, severe disorganization, and pronounced interstitial widening/edema. Because the testicular tissue displayed disorganization of the germinal epithelium, reduced spermatogenic cells, and interstitial edema. Such alterations are indicative of impaired spermatogenesis and potential infertility. Conclusively, quantitative assessment using Johnsen scores revealed a significant dose-dependent reduction in spermatogenic integrity. Control testes exhibited scores consistent with complete spermatogenesis, whereas scores were significantly reduced in the 15 mg/kg group and severely diminished in the 60 mg/kg group (P < 0.05).

## 4 Discussion

The present study demonstrates that sub-chronic oral exposure to polyethylene microplastics (PE-MPs) produces dose-dependent testicular toxicity in male Wistar rats, characterized by impaired mitochondrial bioenergetics, disruption of redox homeostasis, compromised sperm quality, reduced testosterone levels, and marked histopathological degeneration of the testes. These adverse reproductive outcomes were consistently associated with measured inhibition of tricarboxylic acid (TCA) cycle enzymes and electron transport chain (ETC) complexes, alongside quantified increases in oxidative and nitrosative stress markers and depletion of endogenous antioxidant defenses. Taken together, the data identify mitochondrial oxidative phosphorylation impairment and redox imbalance as central, experimentally supported features of PE-MP-induced male reproductive toxicity. These findings echo growing global concerns about the reproductive toxicity of microplastics, especially in the context of chronic environmental exposure, and to our knowledge, this study provides one of the first *in vivo* demonstrations linking PE-MP exposure to coordinated impairment of testicular mitochondrial bioenergetics, redox imbalance, and reproductive dysfunction. The 15 and 60 mg·kg^−1^ oral doses were selected to match previously published sub-chronic PE-MP rodent studies and thereby permit direct mechanistic comparison (Farag et al., 2023; Pa et al., 2020; Kehinde et al., 2025). These doses are higher than typical environmental body burdens reported in humans and wildlife, for example, recent human stool surveys report median microplastic abundances of a few particles per gram of feces, and human blood studies detect MPs at the level of a few particles per milliliter (or in the low-to-mid ng·mL^−1^ range, depending on analytical method). Field surveys in fish and invertebrates commonly report microplastic counts on the order of 0.03–2 items·g^−1^ wet weight, with large spatial and methodological variability (Refosco et al., 2025; Lee et al., 2024; Hartmann et al., 2024; Brits et al., 2024; Porter et al., 2023). Given this disparity, the doses used here are best interpreted as supra-environmental challenge doses chosen to elicit measurable, dose-dependent biochemical and histopathological responses within a practical experimental timeframe and probe mechanisms (mitochondrial and redox disruption) that may operate at lower exposures.

A principal finding of this study is the significant suppression of key mitochondrial metabolic enzymes in testicular tissue following PE-MP exposure. Specifically, the activities of citrate synthase (CS), isocitrate dehydrogenase (IDH), and succinate dehydrogenase (SDH) were significantly reduced, together with pronounced inhibition of, ETC complexes I-IV. Because these enzymes and complexes were directly assayed in isolated testicular mitochondria, the observed reductions constitute direct evidence of mitochondrial bioenergetic impairment, rather than inferred dysfunction. CS activity is widely used as a functional marker of mitochondrial content and integrity (Galgani et al., 2012). Its dose-dependent reduction in PE-MP-exposed rats therefore suggests compromised mitochondrial functional capacity within testicular cells. Concurrent inhibition of IDH implies reduced generation of NADH, limiting electron supply to the, ETC, while SDH inhibition disrupts both the TCA cycle and Complex II-mediated electron transfer. The combined inhibition of, ETC complexes I-IV further demonstrates that PE-MP exposure impairs electron transport across multiple sites rather than selectively targeting a single complex. These complexes serve as key entry points for electrons derived from NADH and FADH_2_. A severe reduction in their activities suggests that electron transfer is significantly impeded, leading to reduced proton pumping across the inner mitochondrial membrane and, consequently, decreased ATP synthesis. As a result of this inefficiency, the testicular mitochondrial electrons may have been prematurely leaked to oxygen, enhancing the generation of ROS, a hallmark of oxidative stress. This oxidative imbalance is particularly detrimental in the testes, where high metabolic activity supports spermatogenesis. Although mitochondrial respiration rates, ATP production, and membrane potential were not directly measured, the simultaneous suppression of multiple TCA cycle enzymes and ETC complexes provides strong biochemical evidence for reduced oxidative phosphorylation efficiency. Similar patterns of enzyme inhibition have been associated with diminished mitochondrial ATP output and increased electron leakage in other toxicological models (Brand and Nicholls, 2011; Bhattacharya and Keating, 2012). Given the high energetic demands of spermatogenesis and steroidogenesis, such mitochondrial inefficiency is sufficient to plausibly contribute to impaired testicular function.

Thus, chronic low-level exposure to PE-MPs may not only impair testicular mitochondrial function but also promote a pro-inflammatory environment within the testicular microenvironment. Mechanistically, the sequence of events observed in this study is consistent with a cascade of mitochondrial-driven cellular disruptions. The mitochondrial bioenergetic defects observed were accompanied by a pronounced disruption of testicular redox balance, as demonstrated by measured increases in oxidative and nitrosative stress markers and quantified depletion of antioxidant defenses. Lipid peroxidation, assessed by malondialdehyde (MDA) levels, was significantly elevated in PE-MP-treated rats, indicating oxidative damage to cellular and mitochondrial membranes. This finding is particularly relevant in testicular tissue and spermatozoa, which are enriched in polyunsaturated fatty acids and therefore highly susceptible to peroxidative injury (Aitken, 2017). At the same time, the activities of superoxide dismutase (SOD), catalase (CAT), and glutathione S-transferase (GST), as well as levels of reduced glutathione (GSH), were significantly reduced. Because these antioxidant systems act in a coordinated manner to neutralize superoxide, hydrogen peroxide, and secondary lipid peroxides, their concurrent depletion reflects a measured collapse of endogenous antioxidant capacity, rather than isolated enzymatic inhibition. This redox imbalance is consistent with enhanced mitochondrial ROS generation secondary to, ETC dysfunction, although ROS production itself was not directly quantified. Nitric oxide (NO) levels were also significantly elevated following PE-MP exposure. While NO plays physiological roles in the testes, excessive NO can contribute to nitrosative stress and cellular injury (Barboza et al., 2020). Although peroxynitrite formation was not directly assessed, the concurrent elevation of NO and oxidative stress markers supports the possibility of enhanced nitrosative damage under PE-MP exposure, a mechanism reported in related reproductive toxicity models (Aitken and Baker, 2020; Barboza et al., 2020). The observed increase in myeloperoxidase (MPO) activity provides further evidence of inflammatory involvement. MPO activity, although classically associated with neutrophils, has been used as a marker of localized inflammatory oxidative stress in testicular tissue and male infertility contexts (Mottola et al., 2024). Its elevation in this study therefore supports the conclusion that oxidative stress and inflammation are experimentally demonstrable contributors to PE-MP-induced testicular injury. These findings reinforce the view that inflammation is not a bystander but a key player in microplastic-induced reproductive toxicity.

The current study also demonstrates that oral exposure to PE-MPs compromises male reproductive health by disrupting sperm quality. These findings add to the growing body of evidence suggesting that microplastic pollution is not only an environmental concern but also a significant biological threat to reproductive function. The biochemical disturbances described above were accompanied by measured, dose-dependent impairments in sperm viability, motility, concentration, and morphology. These outcomes are consistent with the established dependence of spermatogenesis and sperm maturation on intact mitochondrial function and redox balance. Sperm motility, in particular, relies on ATP generated by mitochondrial oxidative phosphorylation in the midpiece, and the observed, ETC inhibition provides a direct biochemical basis for the motility deficits measured in this study. The significant increase in tail abnormalities (coiled, bent, and dwarf tails) and acrosomal defects was quantified through morphological analysis. While ultrastructural or molecular analyses of axonemal or acrosomal proteins were not performed, oxidative stress is known to disrupt microtubule assembly, membrane remodeling, and cytoskeletal integrity during spermiogenesis (Zhou et al., 2023; Jin et al., 2021). Thus, the sperm abnormalities observed here are consistent with, but do not directly prove, oxidative and mitochondrial mechanisms. Importantly, the conclusions drawn are limited to the experimentally measured sperm defects and their plausible association with the documented biochemical disturbances. The marked suppression of serum testosterone observed in PE-MP-exposed rats highlights a potential endocrine-disrupting effect of PE-MPs. Serum testosterone levels were significantly reduced in PE-MP-exposed rats, providing direct evidence of endocrine disruption. Testosterone synthesis in Leydig cells is known to be highly dependent on mitochondrial integrity, particularly for cholesterol transport and steroidogenic enzyme function. Although the expression of steroidogenic acute regulatory (StAR) protein or steroidogenic enzymes was not measured in this study, mitochondrial dysfunction and oxidative stress are well-established inhibitors of Leydig cell steroidogenesis (Dubey et al., 2022; Sofikitis et al., 2008). Therefore, the observed testosterone suppression is consistent with the measured mitochondrial and redox impairments but should not be interpreted as definitive proof of a specific molecular defect. The reduction in testosterone levels coincided with significant decreases in testis, epididymis, and seminal vesicle weights, all of which were directly measured. These changes are consistent with androgen deprivation-associated atrophy of reproductive organs (Sofikitis et al., 2008). Histopathological examination further revealed dose-dependent degeneration of seminiferous tubule architecture, including germ cell loss, epithelial disorganization, and interstitial edema. While quantitative Johnsen scores were not reported, the described histological features are well-recognized morphological correlates of impaired spermatogenesis and hormonal insufficiency (Johnsen, 1970; Li et al., 2021; Ebrahim et al., 2024). Although blood-testis barrier integrity and apoptotic signaling were not directly assessed, the structural degeneration observed is consistent with oxidative and inflammatory injury reported in microplastic exposure studies targeting reproductive tissues (Jin et al., 2021; Li et al., 2021; Wang et al., 2025). These interpretations are therefore presented as supported associations, rather than direct mechanistic demonstrations. The present findings also align with a growing body of evidence demonstrating that microplastics induce male reproductive toxicity through oxidative stress-related mechanisms (Dubey et al., 2022; Lu et al., 2023; Barboza et al., 2020; Jin et al., 2021; Wang et al., 2025; Hou et al., 2021). Importantly, while several studies have reported sperm abnormalities, hormonal disruption, and oxidative stress following exposure to polystyrene or mixed microplastics, fewer have focused on polyethylene microplastics despite their environmental predominance (Yong et al., 2020; Campanale et al., 2020). By directly measuring mitochondrial enzyme activities and redox markers in testicular tissue, this study extends existing literature by providing biochemical evidence linking PE-MP exposure to impaired testicular mitochondrial oxidative phosphorylation.

A major strength of this study is the integrated evaluation of mitochondrial bioenergetics, redox status, sperm quality, hormonal levels, and histopathology within the same experimental model. However, the PE-MPs doses used in this study exceed typical environmental exposure levels for humans. The selected concentrations provide a controlled way to uncover early biochemical, mitochondrial, and histological disruptions that would be difficult to detect at trace environmental levels in a short-term animal study.

## 5 Conclusion

In conclusion, this study provides experimentally supported evidence that polyethylene microplastics disrupt male reproductive function through measured impairment of mitochondrial oxidative phosphorylation and redox homeostasis in testicular tissue. The resulting oxidative and inflammatory stress is associated with compromised sperm quality, testosterone suppression, and structural degeneration of the testes. While certain downstream mechanisms remain inferential, the data firmly establish mitochondrial dysfunction and redox imbalance as central features of PE-MP-induced reproductive toxicity, underscoring the need for further mechanistic and translational investigations into the reproductive risks posed by environmental microplastic exposure. These findings underscore the urgent need for public health interventions, stricter environmental regulations, and further research to delineate safe exposure limits and long-term reproductive risks.

## 6 Limitation

This study has important limitations. Mitochondrial respiration, ATP production, membrane potential, ROS generation, and apoptotic signaling were not directly measured, limiting mechanistic resolution. The doses employed exceed typical environmental exposure levels and should therefore be interpreted as mechanistic challenge doses rather than direct predictors of human risk. Future studies incorporating high-resolution respirometry, mitochondrial imaging, molecular analyses of steroidogenic pathways, and chronic low-dose exposure paradigms will be essential to refine mechanistic understanding and environmental relevance. Finally, sperm evaluation relied on visual scoring rather than computer-assisted sperm analysis, which provides more precise kinematic data. Future studies should incorporate CASA and employ lower, chronic dosing for better translational relevance.

## Data Availability

The raw data supporting the conclusions of this article will be made available by the authors, without undue reservation.
